# Supplementary material for: Development of models for predicting Torsade de Pointes cardiac arrhythmias using perceptron neural networks
Source: BMC Bioinformatics. 2017 Dec 28;18(Suppl 14):497. doi: 10.1186/s12859-017-1895-2 (PMC5751783; doi:10.1186/s12859-017-1895-2)
Supplement: Supplementary file 2 — Distribution of NMR chemical shifts for carbon (left) and nitrogen (right). (DOCX 363 kb) [file 12859_2017_1895_MOESM2_ESM.docx]

**Materials and Methods:**


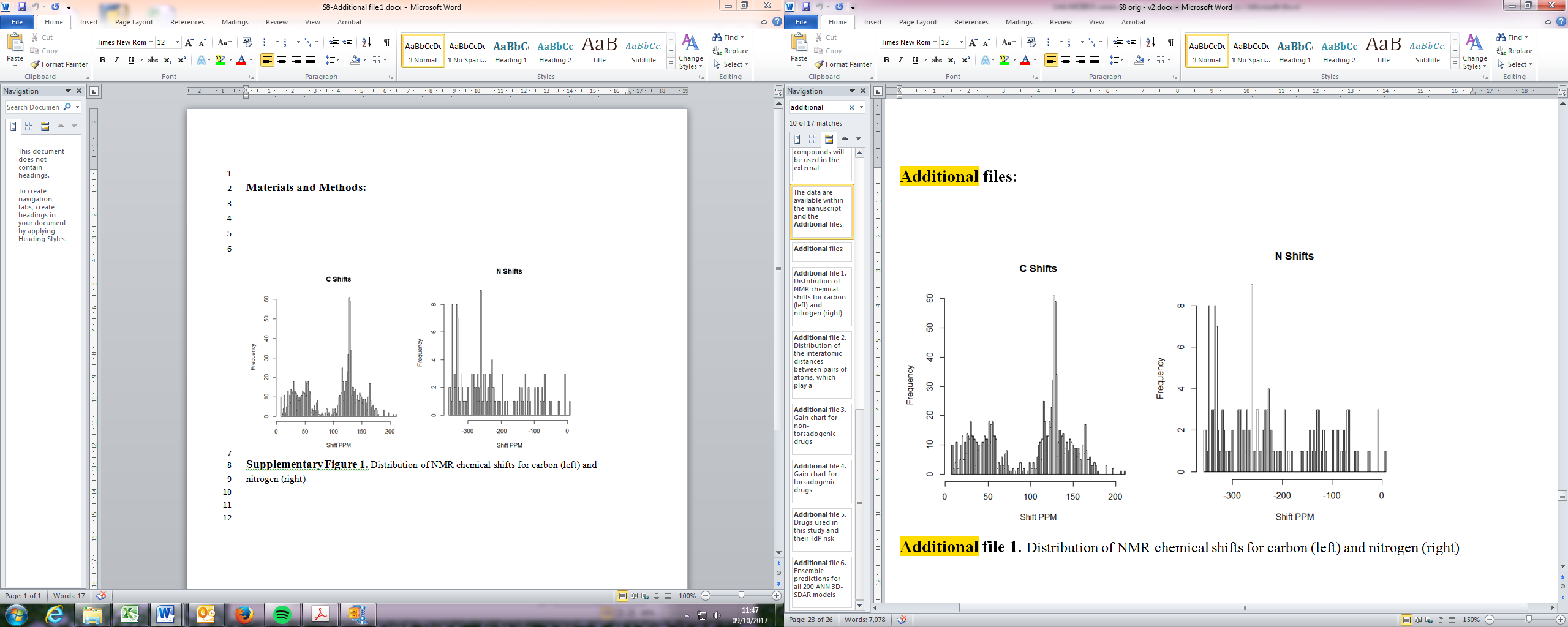


**Figure S1.** Distribution of NMR chemical shifts for carbon (left) and nitrogen (right)
