# Supplementary material for: Development of models for predicting Torsade de Pointes cardiac arrhythmias using perceptron neural networks
Source: BMC Bioinformatics. 2017 Dec 28;18(Suppl 14):497. doi: 10.1186/s12859-017-1895-2 (PMC5751783; doi:10.1186/s12859-017-1895-2)
Supplement: Supplementary file 5 — Gain chart for non-torsadogenic drugs. (DOCX 48 kb) [file 12859_2017_1895_MOESM5_ESM.docx]

**Materials and Methods:**

**Figure S3.** Gain chart for non-torsadogenic drugs
