# Supplementary material for: Development of models for predicting Torsade de Pointes cardiac arrhythmias using perceptron neural networks
Source: BMC Bioinformatics. 2017 Dec 28;18(Suppl 14):497. doi: 10.1186/s12859-017-1895-2 (PMC5751783; doi:10.1186/s12859-017-1895-2)
Supplement: Supplementary file 6 — Gain chart for torsadogenic drugs. (DOCX 112 kb) [file 12859_2017_1895_MOESM6_ESM.docx]

**Materials and Methods:**

Gains Chart - Response/Total Response %

Baseline

Cumulative MLP

10

20

30

40

50

60

70

80

90

100

Percentile

0

1

2

3

4

5

6

7

8

9

10

11

12

13

14

15

16

Gain

**Figure S4.** Gain chart for torsadogenic drugs
